# Acceptance of oclacitinib maleate (Apoquel®) chewable tablets in client-owned dogs with allergic and atopic dermatitis

**DOI:** 10.1186/s12917-022-03210-x

**Published:** 2022-03-17

**Authors:** Marike Visser, Kelly Walsh, Vickie King, Gordon Sture, Laura Caneva

**Affiliations:** 1 Zoetis VMRD, 333 Portage Street, Kalamazoo, MI 49007 USA; 2 Zoetis Belgium SA, Rue Laid Burniat 1, 1348 Louvain-La-Neuve, Belgium

**Keywords:** Apoquel, Allergic dermatitis, Atopic dermatitis, Chewable, Oclacitinib maleate, Dog

## Abstract

**Background:**

The oral acceptance of oclacitinib maleate (Apoquel®) chewable tablets administered twice daily for 7 days at the labeled dose range of 0.4–0.6 mg/kg was evaluated in 121 dogs treated at ten general practice veterinary clinics in the United States.

**Results:**

Dogs that were enrolled in the study were client-owned, ranged from 1 to 14 years of age, weighed 3.7 to 60.7 kg, and required twice daily treatment with Apoquel for allergic or atopic dermatitis for 7 days. One hundred and twenty-one (121) dogs with 1673 total dose administrations successfully completed the study and were included in the data summary. Out of a total number of 1673 administrations, 1533 (91.6%) were accepted voluntarily within 5 min, 134 (8%) were consumed with assistance (with food treats or by pilling) outside of the 5 min offering time and 6 (0.4%) doses were not consumed. The per dose percent acceptance rate for the 14 offered doses showed minimal variation ranging from 89.9 to 93.3%.

**Conclusions:**

Client-owned dogs from the general veterinary patient population that required treatment with oclacitinib found the chewable tablets to be very palatable and no aversion occurred with repeated dosing. Oclacitinib chewable tablets were well tolerated and are a palatable alternative to the film-coated tablet.

## Background

Drug administration compliance can be a challenge with a patient not willing to consume the medication. Developing a palatable formulation enables the owner to administer the medication without the need to hide it in a food or treat item, or to resort to assisted administration (e.g., “manually pilling” the medication), which increases the risk of trauma and stress for both the owner and the pet. Development of a palatable, flavored, chewable formulation that is approved for frequent administration is an approach commonly used by animal health companies to: (1) help pet owners ensure compliance; (2) reduce the stress on the pet and the pet owner associated with administering tableted formulations which can lead to a fracture in the owner-pet bond, and (3) make the overall medication experience more reminiscent of the pleasurable experience of giving the dog a treat. Examples extend across therapeutic categories and include monthly heartworm preventatives (e.g., Heartgard® Plus), nonsteroidal anti-inflammatories (e.g., Rimadyl® Chewables) and antiinfectives (Clavamox® Chewable) among others.

Palatability is a product property, resulting in the product being pleasant or acceptable to taste. In veterinary medicine, this suggests that the product will be voluntarily consumed without the need to hide the product in a treat or food (cheese, bread, etc). Palatability can be evaluated via one of two studies: acceptance or preference. In an acceptance study, the animal is offered the tablet for a set period of time and must consume the whole tablet within that time period (voluntary acceptance). For a product to be voluntarily consumed, the patient must spontaneously consume the product when offered from a bowl, the ground, or from the Owner’s hand [1]. This can be used as a direct measurement of pet compliance. In a preference study, two different dosage forms are offered simultaneously to the animal and the animal can choose which formulation to consume.

While the Food and Drug Administration Center for Veterinary Medicine (FDA CVM) does not provide criteria for a product to be considered palatable; in the European Union (EU), the Committee for Medicinal Products for Veterinary use (CVMP) “Guideline on the demonstration of palatability of veterinary medicinal products” defines a veterinary medicinal product to be palatable and hence allowing a palatability statement to be included in the Summary of Product Characteristics (SPC), if the voluntary acceptance rate in dogs within 2 min is at least 80% [2]. The EU SPC of Apoquel Chewable (authorized in the EU on 13 December 2021) contains the palatability statement “Apoquel tablets are chewable, palatable and readily consumed by the majority of dogs”.^1^

Oclacitinib maleate is a Janus kinase 1 (JAK-1)/signal transducers and activators of transcription (STAT) inhibitor. Apoquel®, is approved globally for the control of pruritus due to allergic dermatitis and the control of atopic dermatitis in dogs. Inhibition of the JAK-1/STAT pathway results in the inhibition of signal transduction of IL-2, IL-4, IL-6, IL-13, and IL-31, pro—inflammatory cytokines associated with clinical signs of allergic and atopic dermatitis [3]. The purpose of this study was to evaluate the acceptance of a chewable tablet containing oclacitinib maleate, a reformulated version of the original Apoquel® film-coated tablet, in client-owned dogs with allergic or atopic dermatitis.

## Methods

This study was conducted as a non-randomized, unmasked, multi-centered clinical trial. Dogs were client-owned, and written informed consent was obtained prior to completion of any study activities. At the time of enrollment, dogs were are least 12 months of age, and weighed a minimum of 3.0 kg (6.6 lbs) and no more than 80.0 kg (175.9 lbs). The dogs were diagnosed with allergic dermatitis or atopic dermatitis and, per the Veterinarian, needed to receive Apoquel twice daily for at least 7 days. Dogs with a diagnosed history or current diagnosis of malignant neoplasia, that were intended for breeding, pregnant or lactating, or had received Apoquel within 7 days prior to enrollment could not be enrolled in the study. A single blood sample for a complete blood count and serum biochemistry was collected to ensure dogs did not have any clinically relevant clinical pathological abnormalities that would require withdrawal from the study.

After enrollment, a 14-day tablet supply was dispensed, however palatability was assessed for the first 7 days of dosing only. The additional 7-day supply was to provide flexibility for scheduling a recheck visit while ensuring patients continued to receive appropriate treatment. All doses were administered in the dog’s normal home environment. If additional medications were being administered on the same day as oclacitinib, the chewable tablets were to be administered first, and the owner was to wait a minimum of 30 min before any medications, treats, supplements or food was administered.

A 5-min timer was started when the dog was given access to the chewable tablet(s). The entire dose was offered by placing the tablet(s) in the dog’s usual empty food bowl or in the palm/fingers of the owner’s hand. A single dose may have been comprised of more than one tablet. The tablet(s) was not broken, crumbled, or crushed except for breaking a tablet in half as necessary to administer the correct dose (e.g., for dogs that require 0.5 or 1.5 tablets per dose). The owner observed the dog carefully during the whole five-minute period to assess whether the total dose was consumed. When the alarm timer sounded (indicating 5 min had elapsed) the owner recorded whether the dose was completely consumed or not. If the dog consumed the complete dose, it was recorded, and no further assessment was needed. If, while dosing the dog (within the five-minute assessment), the owner observed the dog vomiting, regurgitating, or spitting out the tablet, the tablet was re-offered immediately either in the bowl or in the palm/fingers of the owner’s hand. If necessary, a new tablet was offered. If the dose had not been completely consumed within the initial 5 min, the owner administered any unconsumed dose (or portion of the dose) to the dog, using either assisted administration (placing the dose in the dog’s mouth or in the back of the dog’s throat and closing the dog’s mouth until the dose has been swallowed) or the dose may have been administered with food, treat or water. This additional offering ensured that the dog received the appropriate treatment for the underlying condition.

### Data summaries

Data summaries were calculated (SAS/STAT User’s Version 9.4, SAS Institute, Cary, NC). Hypothesis tests were not conducted for this study.

To evaluate overall voluntary acceptance, defined as the percentage of acceptance for the entire period of the study (7 days) where the full dose of the product was voluntarily accepted within the 5-min time period (Eq. 1).

1 $$\frac{Number\ of\ voluntary\ acceptance\ }{Number\ of\ all\ dosings}X\ 100$$

The percentage of doses administered to the dogs via ‘assisted administration (pilling) or treat’ past the 5-min voluntary intake period, was calculated (Eq. 2).

2 $$\frac{Number\ of\ dosings\ administered\ as\ as sisted\ administration\ (pilling)\ or\ treat}{Number\ of\ all\ dosings}X\ 100$$

The percentage of doses that could not be administered to the dogs even with ‘assisted administration (pilling) or treat’, was calculated (Eq. 3).

3 $$\frac{Number\ of\ dosings\ that\ could\ not\ be\ administered}{Number\ of\ all\ dosings}X\ 100$$

The percentage of dogs that voluntarily accepted the dose at all 14 administrations, 13 administrations, 12 administrations etc. was also calculated.

Frequency distributions of breed, sex, and spayed/neutered were calculated. Descriptive statistics for age and initial body weight (mean, standard deviation, minimum and maximum) were calculated.

## Results

A total of 121 dogs were enrolled in the study. Demographics are reported in Table 1. Out of a total number of 1673 administrations, 1533 (91.6%) were accepted voluntarily within 5 min, 134 (8%) were consumed with assistance (with food treats or by pilling) outside of the 5 min offering time and 6 (0.4%) doses were not consumed (Table 1). Dogs did not appear to develop an aversion to the tablets over time, the 5-min acceptance was 93.3% for the first dose and 91.6% for the last dose. The 5-min acceptance at each dosing ranged from 89.9–93.3% (Table 1).

**Table 1:** Demographics of enrolled dogs

Breed	Age (yrs)	Weight (kg)	Sex	
Purebred *n* = 71; Crossbred *n* = 50	1–14	3.7–60.7	Female (*n* = 53)	spayed *n* = 50; intact *n* = 3
			Male (*n* = 68)	neutered *n* = 57; intact *n* = 11

**Table 2:** Summary of Average Acceptance

Acceptance Rate	Total Number	Overall Rate
Overall Voluntary Acceptance within 5 min	1533	91.6%
Overall Consumption with Assistance^a^	134	8.0%
Overall Non-consumption	6	0.4%

^a^ If the dog did not fully consume the offered dose within 5 min, the remaining tablet(s) were administered in food, treat or by pilling

**Table 3:** Average voluntary acceptance within the 5-min assessment window at each dose assessment

Day	AM			PM		
	Number Consumed	Number of Doses	Voluntary Acceptance Rate (%)	Number Consumed	Number of Doses^a^	Voluntary Acceptance Rate (%)
0	111	119^a^	93.3	110	121	90.9
1	109	120^a^	90.8	109	120^b^	90.8
2	111	120^b^	92.5	112	120^b^	93.3
3	111	120^b^	92.5	110	119^b^	92.4
4	110	119^b^	92.4	108	119^b^	90.8
5	107	119^b^	89.9	107	119^b^	89.9
6	109	119^b^	91.6	109	119^b^	91.6

^a^Data point removed due to Owner mis-dosing (e.g., using pill pocket, not dosing) during the 5-min voluntary acceptance assessment

^b^Dog removed from study due to adverse health event

A summary of the number and percent of times the dog accepted all 14 doses down to those dogs that did not accept any of the tablet(s) within the voluntary acceptance window of 5 min is reported in Table 1. Ninety-nine dogs (81.8%) consumed all 14 doses voluntarily within 5 min, while 5 dogs (4.1%) did not voluntarily consume any of the offered doses within 5 min.

**Table 4:** Number of Times a Dog Voluntarily Consumed the Dose within 5-min

Number of Times Dose Fully Consumed	All	
	n	%
**0**	5	4.1
**1**	1	0.8
**3**^a^	1	0.8
**4**	1	0.8
**6**	2	1.7
**7**^b^	1	0.8
**8**	2	1.7
**9**	2	1.7
**10**	1	0.8
**11**^c^	1	0.8
**13**	5	4.1
**14**	99	81.8
**All**	121	100.0

^a^Dog removed due to adverse event after 3 doses

^b^Dog removed due to adverse event after 7 doses

^c^Owner mis-dosed 3 times during the 5-min voluntary assessment window

Nine abnormal health events were observed in 6 dogs during the study. Adverse events included gastrointestinal upset (diarrhea, vomiting, and flatulence), lethargy, and one case of joint pain and lameness. Two dogs were withdrawn from the study due to an abnormal health event, one case of diarrhea and one case of an acute exposure. The enrolled dog, and potentially her housemate, consumed 50 tablets after gaining access to where the chewable tablets were stored. Follow-up examination and clinical pathology results did not report any adverse health events due to the acute exposure.

## Discussion

The overall voluntary acceptance within 5 min of the offering of oclacitinib chewable tablets administered twice daily for 7 days was 91.6% of 1673 dosing events in 121 client-owned dogs with allergic or atopic dermatitis. The per dose percent acceptance rate for the 14 offered doses showed minimal variation ranging from 89.9 to 93.3%, indicating that no aversion occurred with repeated dosing. There is no research regarding the impact of dosing compliance on owner satisfaction and a patient’s response to treatment. In a human study assessing the compliance of patients who took their lipid-lowering medication reports that patients who received 75% or more of their prescribed medication reduce their risk of coronary heart disease from any cause by a third compared to those who took their medication less than 75% of the time [4]. In veterinary medicine, compliance is further complicated if the patient is reluctant to consume the medication. The use of palatable formulations therefore helps to improve compliance by decreasing stress for both the owner and their pet and strengthening the human-animal bond while ensuring the animal receives the appropriate treatment.

## Data Availability

No raw data beyond summarized tables in the manuscript will be provided. The datasets generated and analyzed during the current study are not publicly available due to its proprietary nature, but are available from the corresponding author on reasonable request.

## Abbreviations

CVMP: Committee for Medicinal Products for Veterinary use
EU: European Union
FDA CVM: Food and Drug Administration Center for Veterinary Medicine
JAK-1/STAT: Janus kinase/signal transducers and activators of transcription
Kg: Kilogram
Lbs: pounds
SPC: Summary of Product Characteristics

## Footnotes

1
https://www.ema.europa.eu/en/documents/product-information/apoquel-epar-product-information_en.pdf
